# Effects of a Virtual Reality Reaction Training Protocol on Physical and Cognitive Skills of Young Adults and Their Neural Correlates: A Randomized Controlled Trial Study

**DOI:** 10.3390/brainsci14070663

**Published:** 2024-06-29

**Authors:** Andrea Casella, Camilla Panacci, Merve Aydin, Stefania Lucia, BiancaMaria Di Bello, Francesco Di Russo

**Affiliations:** 1 Department of Movement, Human and Health Sciences, University of Rome “Foro Italico”, 00135 Rome, Italy; a.casella1@studenti.uniroma4.it (A.C.); m.aydin@studenti.uniroma4.it (M.A.); slucia@sissa.it (S.L.); b.dibello@studenti.uniroma4.it (B.D.B.); 2 Santa Lucia Foundation IRCCS, 00179 Rome, Italy; dottoressacamillapanacci@gmail.com; 3 Neuroscience Area, Scuola Internazionale Superiore di Studi Avanzati (SISSA), 34136 Trieste, Italy

**Keywords:** virtual reality, physical performance, cognitive performance, cognitive–motor training, event-related potential, task preparation

## Abstract

Increasing evidence shows that virtual reality (VR) training is highly effective in cognitive and motor rehabilitation. Another modern form of training is cognitive–motor dual-task training (CMDT), which has been demonstrated to rapidly improve physical and cognitive functions in real environments. This study aims to test whether a VR-based CMDT protocol can be used for motor and cognitive skill enhancement in young, healthy subjects. For this aim, 24 university students participated in a randomized control trial. The experimental group participated in a 5-week virtual reality reaction training (VRRT), performing 30 min sessions once a week. The control group did not receive any training but was tested twice with the same measures and temporal distance as the experimental group. Before and after the intervention, motor, cognitive, and electrophysiological measures were assessed. The results showed that following VRRT, the response time for both physical and cognitive tests was improved by about 14% and 12%, respectively, while the control group did not show significant changes. Moreover, electrophysiological data revealed a significant increase in anticipatory motor readiness in premotor brain areas in the experimental group only; however, cognitive top–down control tended to be increased in prefrontal areas after VRRT. This training protocol in a VR modality seems to be as effective as other CMDT methodologies carried out in a real modality. Still, it has the advantages of being more flexible and more user-friendly compared to standard training. The VRRT’s efficacy on physical and cognitive functions indicates that virtual reality applications can be used by the young population, not only for entertainment purposes but also in the form of cognitive–motor training.

## 1. Introduction

Virtual reality (VR) is defined as the “interactive visualization of virtual images enhanced by special processing and nonvisual display modes: to convince participants that they are immersed in a synthetic space” [1]. VR technology has advanced significantly in recent years and seems to be among the most intriguing and promising developments in computer graphics. Specifically, it uses interactive devices to artificially generate an environment as close to the real world as possible, in which users can interact with its tridimensional entities [2,3,4]. One of the greatest values of VR is that it can be used as a training methodology to increase cognitive and motor performance through highly immersive scenarios [5]. VR-based training presents undeniable advantages compared to real training for many reasons: Any details of the environment can be fully manipulated, always keeping it safe and controlled [6,7]. The physical and cognitive load can be finely graduated to be suited to the individual and increased or decreased according to the training goals [6,8,9]. VR training allows multimodal and immediate feedback on performance that facilitates learning [8] and helps the trainer modulate the task. In addition, immediate response-related feedback may reduce uncertainty and anxiety about performance [10]. Finally, VR training can be executed at home with remote supervision.

In recent years, the scientific literature has focused on VR training through a combination of technologies since it seems to offer an excellent improvement in specific health needs, including cognitive enhancement and rehabilitation, particularly in the area of cognitive impairment [11]. Not surprisingly, several studies have confirmed that specific VR training can induce states of relaxation and positively stimulate cognitive and executive functioning. These benefits are provided by the ability to immerse users in 360 degrees within the desired scenario. These scenarios, unlike real life, may consist of many stimuli and distractors with which the user must interface to achieve one or more specific goals [12]. Hence, among the main purposes of VR training, the facilitation of cognitive modeling and enhancement through virtual environments specifically designed to simulate both the external and internal worlds assumes centrality. Previous studies have suggested that cognitive training in VR may improve not only people’s cognitive functions and emotional experiences [11,12,13] but also their daily living skills [14,15]. Thus, VR cognitive training can stimulate the brain to improve its cognitive functions. Among the main positive effects on relevant neurobiological mechanisms, cognitive plasticity and neuroplasticity are the most relevant [16,17,18]. Considering these findings, it seems clear that, currently, VR is considered a supportive tool for the treatment of some neuropsychological and psychological disorders (i.e., anxiety, depression, and cognitive decline) and, in specific cases, for post-stroke rehabilitation [18,19].

Another modern form of valuable training is cognitive–motor dual-task training (CMDT), which simultaneously combines physical and cognitive exercises. Many studies on CMDT have demonstrated its effectiveness in rapidly improving both physical and cognitive functions in both old [20] and young people [21]. Studies investigating the neural bases of the effects of CMDT indicated that it may stimulate anticipatory brain function in prefrontal and premotor areas, allowing for more predictive neural resources to be deployed in relation to action anticipation in sensorimotor cognitive tasks [20,21]. Studies on old people used CMDT to counteract age-related cognitive and motor decline. Studies on young people focused on high-level athletes, and CMDT was used to improve sport performance. However, while the aforementioned areas of VR application are commendable and worthy of special attention, there seems to be a significant gap in the literature about the physical and cognitive benefits that can be experienced from specific VR training in the healthy population. Commercial VR systems are mainly used by young adults for entertainment, but this technology may potentially offer numerous benefits in different areas of daily life, such as increased performance in school, work, and sports [22,23].

This paper aimed to test a VR training protocol on healthy people, not only to expand our knowledge in VR research but also to verify whether the numerous benefits offered by VR training in patients could be useful in healthy populations. To maximize the benefit offered by VR on cognitive and motor performance, we used CMDT as a training modality for use in VR. Specifically, we expect that (such as for CMDT in the real environment), VR-based CMDT could also improve physical and cognitive performance in young, healthy people. To do this, we used a device and software currently available in stores. If effective, such training could offer numerous benefits in different areas of daily life, increasing performance in school, work, and sports. Indeed, in these areas, cognitive functions play a crucial role in achieving successful outcomes [24,25]. We also aimed to test if VR-based CMDT could affect anticipatory brain processing. Specifically, given the results obtained by Lucia et al. [20,21], the main objective of this research project was to investigate the influence of VRRT on specific ERP components. Specifically, we hypothesized that, following the training, the experimental group would report significant benefits in the components prefrontal negativity (pN) and Bereitschaftspotential (BP), which reflect cognitive anticipation and motor preparation, respectively. Similarly, given the characteristics of VRRT, we hypothesized that the experimental group would report significant benefits in motor testing compared with the control group.

To carry this out, we used techniques from neurophysiology to study the quick and intricate neural processing that takes place when performing motor and cognitive activities. More precisely, event-related potentials (ERPs) and electroencephalography (EEG) enable millisecond-accuracy detection of brain processes. Because of their excellent temporal precision, ERPs have been used to successfully detect the temporal course of cognitive processes, spanning task preparation to motor execution [26]. According to ERP research, certain training regimens might affect the anticipatory brain activity that is necessary for completing difficult sensory–motor cognitive tasks [20,21]. Response discrimination tasks (DRTs), like the Go/No-go paradigm, have been used in these investigations because they strongly engage anticipatory cognitive functions [26,27]. In fact, their attention was drawn to pre-stimulus anticipatory ERP elements, such as prefrontal negativity (pN) and Bereitschaftspotential (BP), which begin to manifest around one second before the beginning of the stimulus. It has been demonstrated that in DRTs, response accuracy is predicted by pN, meaning that the higher the pN, the lower the error rate, and response time (RT) is predicted using BP amplitude, meaning that the greater the BP, the quicker the RT. While the pN, which originates from the inferior frontal gyrus [27], has been linked to response accuracy, proactive cognitive functions like top–down attention and inhibition in the prefrontal cortex for complex tasks [26,27], BP is an anticipatory readiness potential that reflects the excitability of the supplementary motor and cingulate areas and emerges before any voluntary act [28]. Additionally, it has been suggested that these elements provide the neurological foundation of an activation/inhibition (braking/accelerating) cognitive system, a system that influences the speed/accuracy tradeoff by foreseeing and predicting future actions and occurrences [26]. Furthermore, results from CMDT interventions that explored the effect on behavioral performance (RT, accuracy) and investigated the effect of plasticity and compensation on BP and pN in basketball players already provided evidence of the effectiveness of these training protocols [20,21]. According to this literature, we expect that in case of improvement in response time and accuracy, increased BP and pN amplitudes should be correspondingly found.

## 2. Materials and Methods

### 2.1. Participants

Using Cohen’s f statistics to estimate effect size, G*power 3.1.9.2 software was used to determine the sample size. Based on a work with a comparable design and measures [20], we set the predicted effect size f(V) for the current mixed 2 × 2 ANOVA design at 0.31. The α level was set at 0.05, and the intended power $\left(1-\beta \right)$ was set at 0.95 (estimated sample size = 24). Thus, for this study, a total of twenty-four university students—twelve males and twelve females—with a mean age of 24.5 years (SD = 2.5), were enlisted. The following conditions had to be met in order for a participant to be considered for inclusion: normal or corrected-to-normal eyesight, no medical illness or neurological or psychiatric illnesses, and no medication were to be used during the trial session. Before taking part in this study, the participants provided their informed consent in line with the Declaration of Helsinki, with clearance from the University of Rome’s “Foro Italico” local ethics council.

### 2.2. Procedure

In these randomized control trials, the participants were pseudo-randomly assigned to two groups of 12: the experimental (Exp) and the control (Con) group, which were balanced for the daily level of physical activity, measured using the International Physical Activity Questionnaire (IPAQ) [29]. Preliminary *t*-test indicated that the groups did not differ in terms of age, education, and socioeconomic status (t_(23)_ > 1). Both groups participated in two screening test sessions six weeks apart, including physical, cognitive, and electrophysiological measures. During this period, the Exp group performed five 30 min sessions of VRRT, described below, once a week. The control group was screened twice only to control the learning effect of the employed measures.

### 2.3. Screening

#### 2.3.1. Physical Test

To measure the physical performance, a 10 m sprint test was used. For this test, a Witty-SEM device (Microgate, Bolzano, Italy) was placed in front of the standing participant at a 1.5 m height. The display showed a 3-2-1 countdown (number duration 1 s), became black for a random interval from 1 to 5 s, and then turned to green, indicating that the participant should start a 10 m linear sprint as fast as possible. A photocell, connected to the Witty-SEM device and located at the beginning of the 10 m running track, measured the sprint reaction time. Each participant performed three attempts, but only the best reaction time was recorded.

#### 2.3.2. Cognitive Test

**Figure 1:**
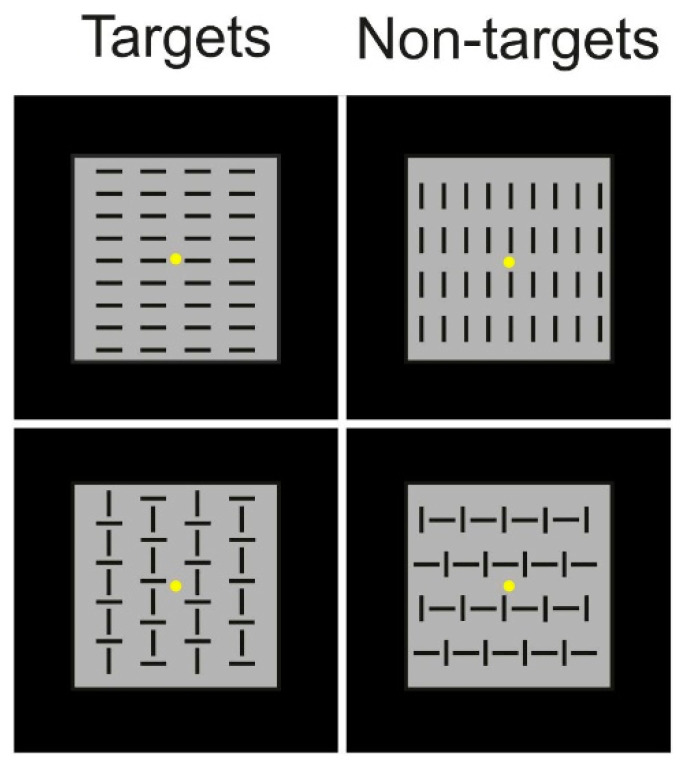
Depiction of the stimuli employed in the cognitive task, distinguishing between target and non-target stimuli. The yellow dots represent the fixation point.

A visuomotor discrimination response task (DRT), commonly referred to as “Go/No-go”, was conducted as part of the EEG recording process. The participants had the EEG cap fitted over their scalps, and then they were tested in a soundproof, dimly lit room. The participants sat in front of a computer screen that was 114 cm away from their eyes, and they touched a response box with their right index finger. A fixation point appeared at the center of the screen on a black background at the beginning of each trial and stayed there the entire time. As shown in Figure 1, four visual stimuli, each with an equal probability (*p* = 0.25) and represented by square configurations measuring 4 × 4° made up of vertical and/or horizontal bars, were randomly shown for 250 ms each. To avoid anticipation and ERP overlaps with either the previous or upcoming stimuli, the intervals between stimulus onsets were spaced out by one to two seconds. Two predetermined target stimuli were shown to the participants (*p* = 0.5), and they were told to hit the button as soon as they appeared. Non-target stimuli were not to be responded to (*p* = 0.5). The instructions placed equal emphasis on precision and quickness. The presentation order of the stimuli was not predetermined. There were gaps of two minutes between each run. Ten runs were carried out in all, resulting in roughly 400 trials for every stimulus category in a 25–30 min period.

#### 2.3.3. Behavioral Data

The average response time (RT) for accurate trials was computed. Accuracy was determined by combining the percentage of commission errors (i.e., responses to non-target stimuli) and omission errors (i.e., missed responses to target stimuli).

#### 2.3.4. EEG Recording and Analysis

EEG recordings were obtained using BrainVision Recorder 1.2 software, receiving signals from three BrainAmp^TM^ amplifiers. Two of them were connected to 64 active scalp electrodes (ActiCap^TM^) andAnalyzer 2.3 software was used for data processing (all by BrainProducts GmbH., Gilching, Germany). The channels were positioned at the following sites: Fp1, Fp2, F3, F4, C3, C4, P3, P4, O1, O2, F7, F8, T3, T4, T5, T6, Fz, Cz, Pz, Oz, FC1, FC2, FC5, FC6, CP1, CP2, CP5, CP6, PO3, PO4, AF3, AF4, F1, F2, F5, F6, FC3, FC4, C1, C2, C5, C6, CP3, CP4, P1, P2, PO7, PO8, FT7, FT8, TP7, TP8, F9, F10, FT9, FT10, T9, T10, P9, P10, Iz, AF7, AF8, and CPz. The scalp electrodes were mounted according to the 10-10. The M1-M2 electrodes were set as references. A Butterworth zero-phase filter (0.01–60 Hz; second order) was used to band-pass filter the amplified EEG signals, which were then stored for offline analysis. The data were digitized at 250 Hz. The third BrainAmp amplifier (E×G type) was used to record the electrooculogram (EOG), which tracked eye movements in bipolar mode. The electrodes were positioned above the outer canthi of the left and right eyes to record the horizontal EOG, while electrodes were put below and above the left eye to capture the vertical EOG. The impedances of the electrodes were kept below 5 KΩ. Analysis of the blink and vertical eye movement artifacts was carried out automatically with Analyzer 2.3’s independent component analysis using the “Meaned Slope” algorithm”. Subsequently, to remove other artifacts due to sweating, muscle tension, and/or heartbeats, periods where amplitudes exceeded the threshold of ±70 μV were excluded from further analysis. EEG data were segmented into 1300 ms epochs, commencing 1100 ms prior to stimulus onset and concluding 200 ms following stimulus onset, in order to assess pre-stimulus activity. A baseline of −1100/−900 ms was applied to the first 200 ms. The target and non-target trials were averaged since the stimulus category was unpredictable before stimulus onset. To establish intervals and electrodes to be used for statistical analysis, the “collapsed localizer” approach was used. The collapsed localizer is the average of all considered ERP data across all groups and conditions, as described in [30]. This approach ensures that the intervals and electrodes selected for analysis are representative and unbiased across different experimental conditions. To choose the analysis interval, the global field power (GFP), which represents ERP spatial variability across all scalp electrodes simultaneously, was computed [31]. For additional analysis, this meant determining the pre-stimulus interval during which the GFP surpassed 70% of its highest value. The GFP-based method identified a range of −300 ms to 0 ms, within which the mean amplitude under all conditions was calculated for statistical analysis. In the interval set by the collapsed localizer, electrodes exhibiting amplitudes greater than 70% of the maximum value were averaged into spatial pools and used for statistical analysis. The medial prefrontal activity of the pN and the medial centro-parietal activity of the BP components were the two main activity foci that were identified. The pre-frontal pool, which included the electrodes Fpz, Fp2, AFz, and AF4 to represent the pN, and the centroparietal pool, which included the electrodes CP2, CPz, C2, and Pz to represent the BP.

### 2.4. Intervention

**Figure 2:**
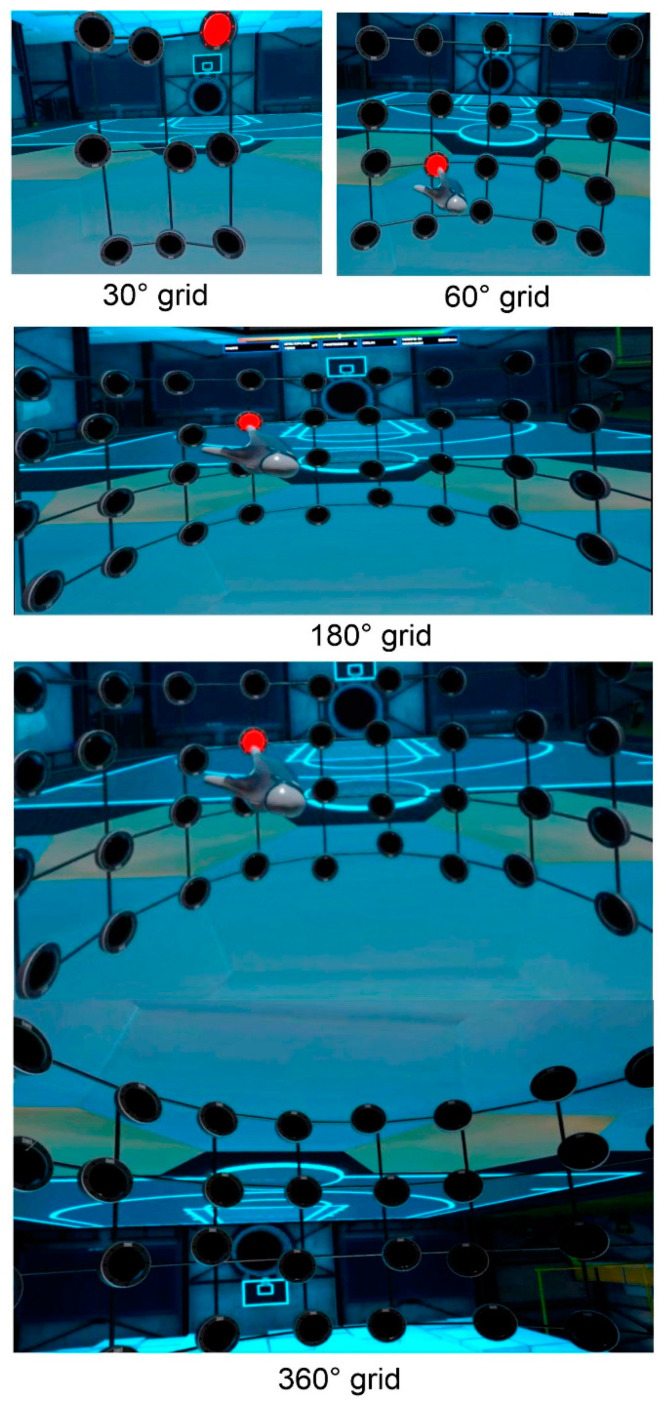
Views of the VR training at the four grades of difficulty. The red circle represents the target stimulus.

The Exp group performed five 30 min sessions (once a week) of VR training using a Meta Quest 2 system (Meta Platforms, Menlo Park, CA, USA) and “Rezzil Player 1.1.1265, Manchester, United Kingdom” software, available in Meta Quest store. This software was chosen because it includes a collection of specific sports training and fulfilled the CMDT requirements since it includes simultaneous motor and cognitive exercises with incremental cognitive and motor loads [20,21,22]. The training used was the “reaction wall”, which requires participants to touch, as quickly as possible, the stimuli that light up in a grid of lights wall in front of him/her. Red and blue stimuli had to be touched with the right and left hand, respectively, and the stimulus position randomly varied. The training has incremental levels of difficulty; at the first level, the stimulus duration (500 ms) and interstimulus interval (1000 ms) were fixed. At the second level, these durations decreased proportionally to the speed of the subject’s response. Each level had four different difficulties grades in terms of the stimuli present in the visual field: (a) a 3 × 3 grid subtending 30° of horizontal visual angle with nine stimulus positions; (b) a 5 × 4 grid subtending 60° with 20 positions; and (c) a 10 × 4 grid subtending 180° with 40 positions; (d) 20 × 4 grid subtending 360° with 40 positions. In the first level, each grid lasted 60 s with a 30 s break in between. In the second level, grids lasted 120 s with 30 s breaks. The four grids visualized by participants are shown in Figure 2.

### 2.5. Statistical Analysis

Shapiro–Wilk’s W test was used to evaluate the assumption of normality for each measure. The results showed that all of the measures were non-significant, indicating that they adhered to normal distributions. Using Levene’s test for equality of variance to assess the homoscedasticity assumption, no sample homoscedasticity violation was found. All measurements were subjected to mixed 2 × 2 ANOVAs with Group (Exp vs. Con) and Time (Pre-test vs. Post-test) as the factors after these preliminary tests. The reported effect sizes were expressed as partial eta squared (η_p_^2^). We used Bonferroni correction for post hoc comparisons. A total of 0.05 was chosen as the alpha level. Statistica 12.0 (StatSoft Inc., Tulsa, OK, USA) was used to perform statistical analyses.

## 3. Results

### 3.1. Physical Test

**Figure 3:**
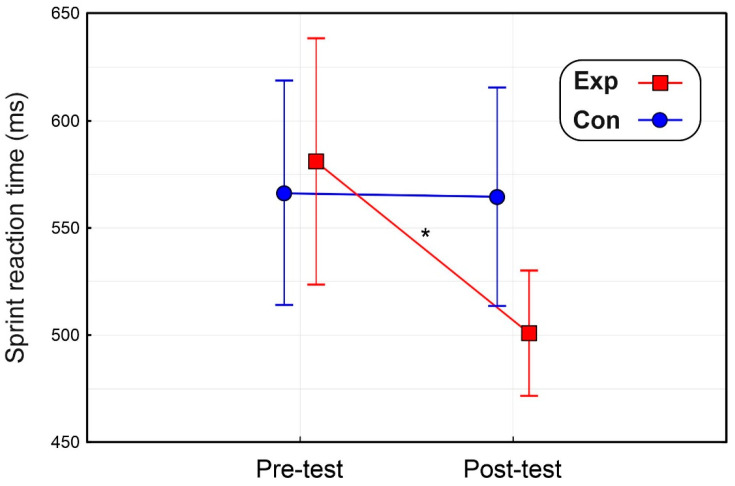
Results of the motor task showing the sprint reaction time. Vertical bars denote 0.95 confidence intervals. * *p* < 0.05.

The ANOVA on the sprint reaction time showed that the effect of Group (F < 1) and Time (F_(1,22)_ = 1.6, *p* = 0.218, η_p_^2^ = 0.071) were not significant, but was significant the Group × Time interaction (F_(1,22)_ = 4.4, *p* = 0.048, η_p_^2^ = 0.167). As shown in Figure 3, post hoc comparisons indicated that while in the Con group, the sprint reaction time did not change (pre-test = 565 ms ± 105, post-test = 562 ms ± 103), in the Exp group, the reaction time in the post-test (501 ms ± 97) was shorter (*p* = 0.026) than the pre-test (580 ms ± 120).

### 3.2. Cognitive Test

**Figure 4:**
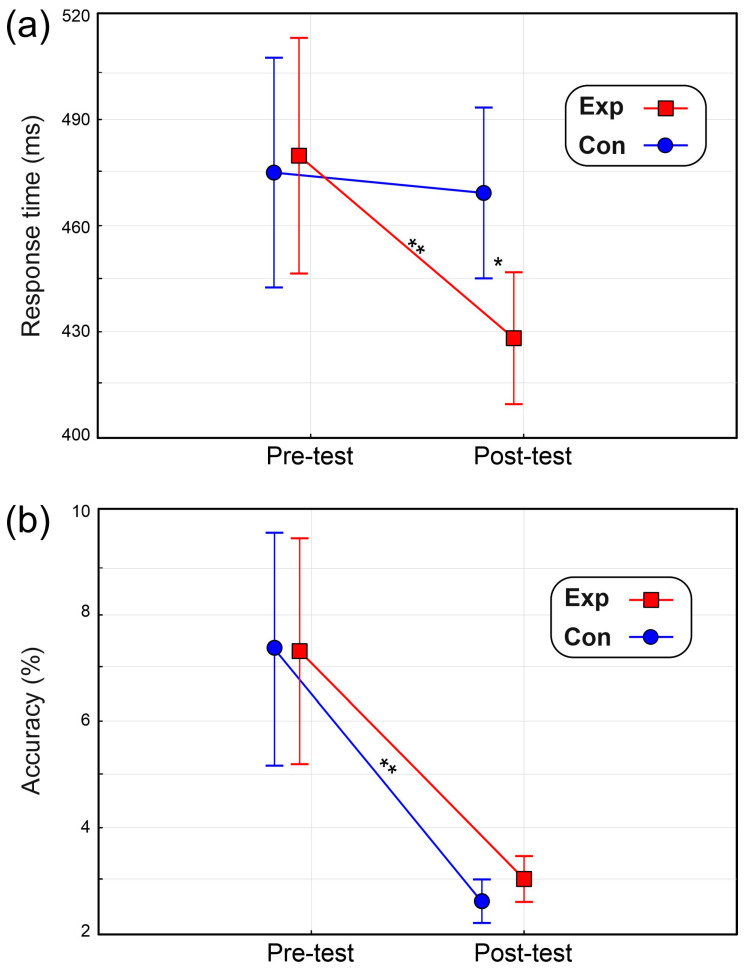
Results in the cognitive task for (**a**) response time and (**b**) accuracy (error percentage). Vertical bars denote 0.95 confidence intervals. * *p* < 0.05, ** *p* < 0.01.

The ANOVA on the RT showed a non-significant effect of Group (F_(1,22)_ = 3.7, *p* = 0.067, η_p_^2^ = 0.145) and a significant effect of Time (F_(1,22)_ = 8.5, *p* = 0.008, η_p_^2^= 0.282). However, the Group × Time interaction was significant (F_(1,22)_ = 6.2, *p* = 0.021, η_p_^2^ = 0.219). As depicted in Figure 4a, post hoc comparisons indicated that while in the Con group, the RT did not significatively change (pre-test = 475 ms ± 99, post-test = 471 ms ± 97), and in the Exp group, the RT in the post-test (429 ms ± 103) was shorter (*p* = 0.005) than the pre-test (482 ms ± 91) and was also shorter (*p* = 0.018) than the post-test on the Con group.

ANOVA on accuracy revealed no significant effects of Group (F_(1,22)_ = 1.6, *p* = 0.215, η_p_^2^ = 0.074). The effect of Time was significant (F_(1,22)_ = 19.6, *p* < 0.001, η_p_^2^ = 0.889), indicating fewer errors in the post-test (2.8% ± 0.3) than in the pre-test (7.3% ± 1.5). As shown in Figure 4b, Group × Treatment interaction was not significant (F < 1).

### 3.3. ERP Results

**Figure 5:**
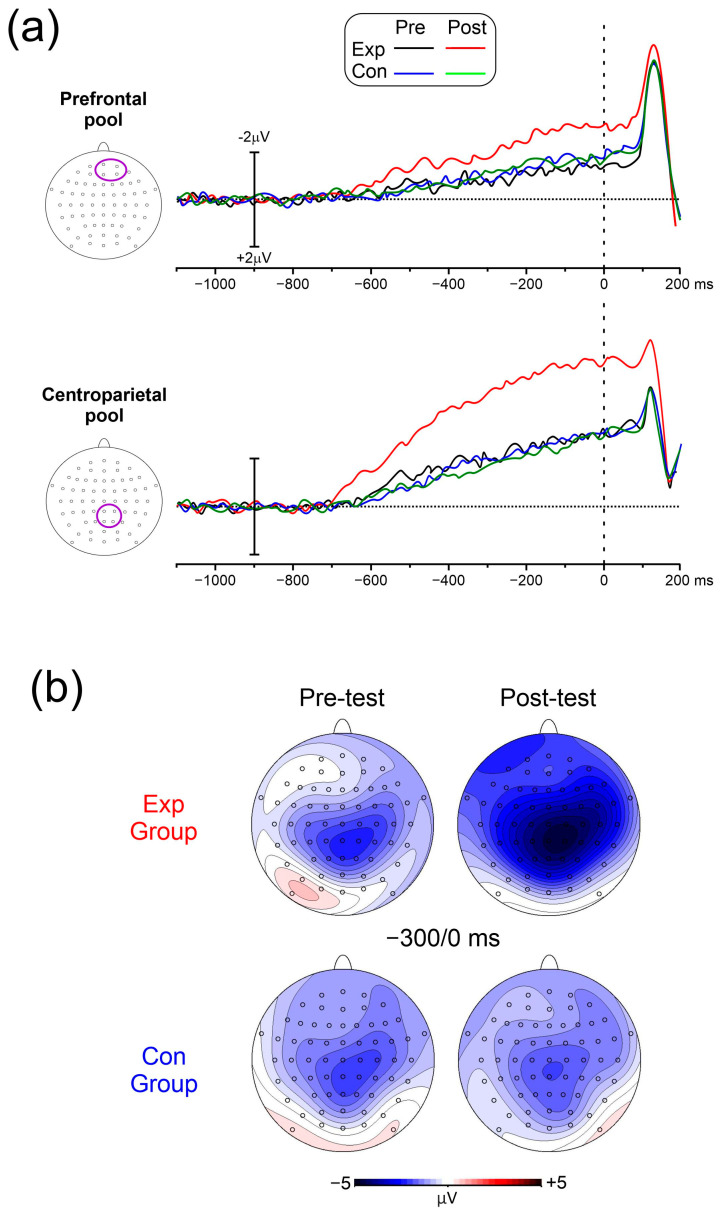
(**a**) Pre-stimulus ERP waveforms at the prefrontal and centroparietal pools. (**b**) Scalp voltage topography in the −300/0 ms time window from a top–flat view.

Figure 5a shows the pre-stimulus ERP waveforms in the four conditions. Figure 5b shows the voltage and topographical distribution in the −300/0 ms interval. The BP is the first detectable activity starting from −700 ms and emerging as slow-rising negativity, reaching its peak at stimulus onset on medial centroparietal sites. The pN initiated between −620 ms and −600 ms and peaked at stimulus onset on medial prefrontal sites.

**Figure 6:**
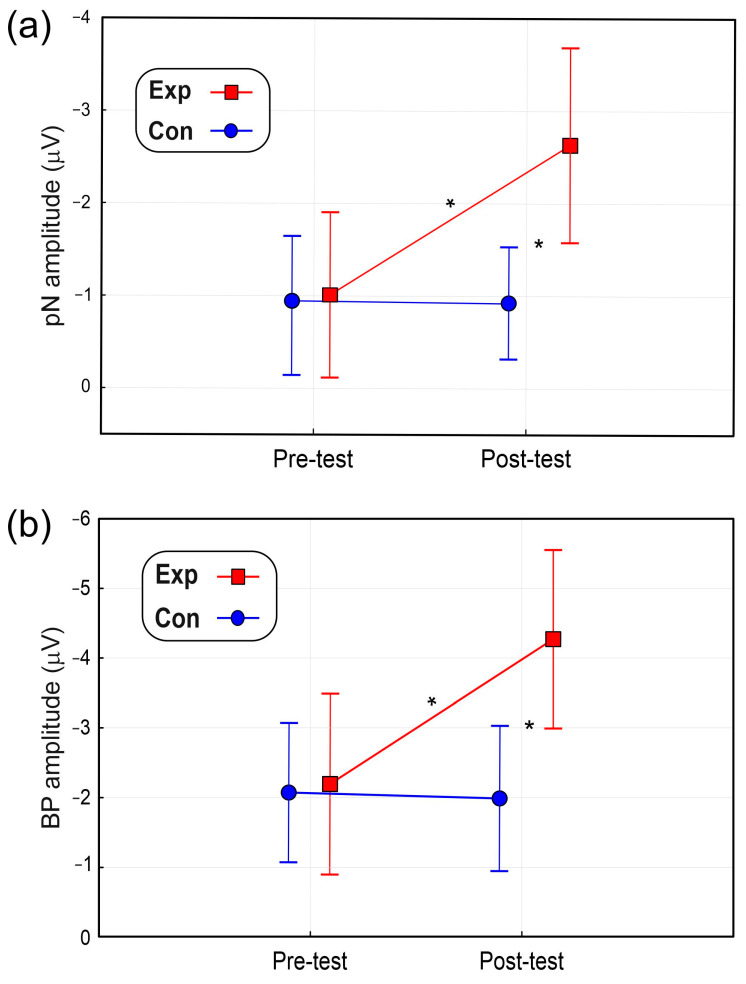
(**a**) pN amplitude. (**b**) BP amplitude. Vertical bars denote 0.95 confidence intervals. * *p* < 0.05.

ANOVA on the pN showed a significant effect of Group (F_(1,22)_ = 5.7, *p* = 0.026, η_p_^2^ = 0.206) and a non-significant effect of Time (F_(1,22)_ = 2.9, *p* = 0.103, η_p_^2^ = 0.116). The Group × Time interaction showed a significant tendency (F_(1,22)_ = 4.2, *p* = 0.052, η_p_^2^ = 0.142). Post hoc comparisons indicated that the pN amplitude in the post-test of the Exp group (−2.63 µV ± 0.84) was larger (*p* = 0.048) than the pre-test (−1.01 µV ± 0.71) and was also larger (*p* = 0.032) than the post-test amplitude of the Con group (0.94 µV, ±0.42). The pN amplitudes of the Con group did not significantly differ from each other. Figure 6a shows this interaction.

ANOVA on the BP showed a non-significant effect of Group (F_(1,22)_ = 1.9, *p* = 0.182, η_p_^2^ = 0.079) and a tendency of the Time effect (F_(1,22)_ = 4.0, *p* = 0.059, η_p_^2^ = 0.153). However, the Group × Time interaction was significant (F_(1,22)_ = 4.5, *p* = 0.045, η_p_^2^ = 0.170). Post hoc comparisons indicated that the BP amplitude in the post-test of the Exp group (−4.30 µV ± 2.71) was larger (*p* = 0.039) than the pre-test (−2.21 µV ± 2.26) and was also larger (*p* = 0.042) than the post-test amplitude of the Con group (1.97 µV ± 0.88). The BP amplitudes of the Con group did not significantly differ. Figure 6b shows this interaction.

## 4. Discussion

In the present study, we tested the effect of a VR-based CMDT protocol (named VRRT) on healthy participants on their performance in physical and cognitive tasks. In addition, we studied the neural correlates of this effect using ERP measures of anticipatory brain processing in the prefrontal (the pN) and premotor (the BP) areas. The results showed that following the proposed VRRT, the response time for both the 10 m sprint and the cognitive task was improved by about 14% and 12%, respectively, in only five weeks, while the control group did not show any significant effect. For VR applications in healthy people, this result is novel since no studies found response time improvements in both motor and cognitive tests after a VR treatment. However, the results are in line with two studies [32,33] comparing the performance in a Stroop task before and after a short session of a VR-based visual training intervention. These studies found that the response time in a Stroop task was shorter after the training. We also confirmed the effectiveness of the CMDT modality in a VR environment with comparable results as found in non-virtual CMDT protocols in the same short amount of time [20,21,34]. However, this result is supported by findings [14] comparing a standard CMDT protocol with the same protocol but conducted in VR on a group of adults with mild cognitive impairments (MCI). This study reported significant cognitive (e.g., cognitive flexibility, attention, and information processing) and motor benefits (e.g., increased gait speed) following both training protocols. However, VR training yielded greater benefits in terms of global cognition and instrumental activities of daily living. Related results were found in a study comparing VR-based cognitive training and aerobic and resistance exercise training protocols [35]. The response accuracy in the cognitive task improved in both groups, likely indicating a learning effect. The lack of accuracy effect may be due to the low cognitive demand required by the training that privileged speed and not response accuracy. Indeed, in the tested VRRT, the distinction between two colors to be touched with the right or left hand does not seem to be sufficiently complex to produce significant accuracy benefits in the experimental group. Considering that other CMDT protocols with higher complexity than those present in this study found both response speed and accuracy effects [20,21], future studies could test the effect of training with an increased number and shapes of targets that could be mixed with non-targets and distractors. Such modifications would not only increase the chances of obtaining significant accurate results but also significantly broaden the scope of application for the training. The neural basis of the behavioral performance effects of the tested VRRT can be seen by the ERP analysis, showing that the experimental group only had a significant increase in motor preparation, as evidenced by the BP components amplitude. Given that, following a careful analysis of the literature, no studies were found on the influence of VR training on the brain activity of the general young adult population. However, [21] observed shorter RT and larger BP in a group of young basketball athletes after 5 weeks of real (non-virtual) CMDT. The comparable results found here confirm the efficacy of CMDT even in VR. Furthermore, the result of larger BP and shorter RT is consistent with the results in the literature [26], confirming the association of this motor preparation ERP component with the RT. Concerning the pN component, which reflects attentional and inhibitory preparation for complex tasks, this component showed a trend toward significance with a larger amplitude in the post-test in the experimental group only. This result could indicate that the present VR training, which requires inhibitory and attentional skills to complete the task, may also stimulate these capacities but to a lesser degree, and this effect could be covered by task learning. However, the VR training effect on prefrontal areas confirms previous studies on healthy adults [34] and adults with MCI [14,35,36]. These studies showed the modification of functional near-infrared spectroscopy measures over prefrontal areas after virtual training protocols and suggested an increase in neural efficiency in the VR-trained participants. According to the accelerator/braking system proposal [26], the present training seems to affect the speed/accuracy tradeoff stimulating the accelerator more and the brake less, therefore allowing faster response without limiting accuracy. This pattern of results provides further evidence of the functionality of VR training in positively influencing brain plasticity, and it highlights potential uses of VR in enhancing anticipatory cognitive–motor functions in the healthy population. An explanation of how the tested VRRT protocol enhanced these preparatory brain processes may come from studies on CMDT (reviewed in [37]). VRRT, being a dual task, may produce an over-additive activation of the promotor and prefrontal brain areas that play a key role in managing the concurrent execution of more tasks [38].

Specifically, the effects found raise considerations about the possible application of this training protocol in various daily life contexts, such as for academic, occupational, and sports achievements. In particular, long-standing research [39] and more recent studies [38] emphasized the importance of motor preparation in athletes. These findings are supported by previous electrophysiological studies that found greater cognitive and motor preparation abilities in expert athletes compared to novices [37]. From these studies, it seems evident that motor preparation plays a key role in achieving successful performance. Therefore, future VR training protocols could be designed to allow successful task performance in specific tasks. For example, Ref. [40] highlights the role played by motor preparation while driving vehicles on the road. Indeed, our brain serves multiple functions beyond the control of body movements. It engages in activities such as sensory–motor transformation, comprehension of actions, decision-making processes related to the initiation and execution of actions, as well as the preparation and planning of intricate movements [41,42,43,44]. Notably, Ref. [44] showed that specific neurons in the caudal part of the dorsal premotor cortex activate in response to the onset of movement, others become active during the anticipation of a ‘go’ signal, and others respond to the appearance of instructing stimuli. Therefore, what appears evident to date is that specific VR training protocols can significantly influence brain plasticity, creating new possibilities for non-pharmacologic therapeutic intervention in the pathological population [45] and offering opportunities for cognitive enhancement in the healthy population, such as improving study and work. To increase the validity of the results, further studies should compare VR training with different cognitive and motor training methodologies to identify the optimal training in function of age and work. Finally, the level of perceived embodiment must also be taken into consideration, as it significantly influences performance [46] and depends on the limitations of the VR device used.

This study has the following limitations: (a) due to the limited sample size due to the difficulties in recruiting participants; the results should be confirmed in a large sample; (b) as the sample consisted of students from a university of sports sciences, the sports activities practiced by participants may have influenced the results. Even if we controlled for the physical activity level using IPAQ, we could not control for the effects of the specific sport. (c) Using a commercial application, the training scenarios could only be minimally modified to obtain the desiderated balance between motor and cognitive loads; (d) prolonged use of VR may cause physical discomforts such as nausea, dizziness, or visual fatigue, known as ‘cybersickness’. However, we closely monitored participants and allowed regular breaks during VR training sessions.

## 5. Conclusions

This study tested the efficacy of a virtual reality-based dual-task cognitive–motor training protocol, named VRRT, in improving physical and cognitive performance in healthy individuals. The results showed significant enhancements in response times for motor and cognitive tasks after only five weeks of VRRT, highlighting the potential of VR technology in increasing human performance. In addition, while response accuracy in the cognitive task improved in both groups, indicating a learning effect, the VRRT group showed a significant increase in motor preparation, as evidenced by ERP measures of anticipatory brain processing. These results emphasize the functional benefit of VR training in positively influencing brain plasticity and improving anticipatory cognitive–motor function. Importantly, our study fills a significant gap in the literature by demonstrating the benefits of specific VR training in healthy populations, which has significant implications for various domains such as education, employment, and sports performance. In addition, the observed effects raise intriguing possibilities for the application of VR training protocols in various real-world settings. Future research should explore the comparative effectiveness of VR training with other training methodologies, considering factors such as age and occupation. Additionally, the perceived embodiment within the virtual environment should be further investigated as it influences performance and is contingent on the capabilities of the VR device utilized. Overall, this study contributes to the rising body of evidence supporting the potential of VR technology as a tool for cognitive enhancement and performance optimization, both in clinical populations and among healthy individuals. By harnessing the power of immersive virtual environments, we can support new evidence for non-pharmacological therapeutic interventions and foster cognitive growth and development in various areas of daily life.

## Data Availability

Data will be made available upon request. Data are not publicly unavailable due to the privacy restrictions of the University of Rome Foro Italico.
